# A combined role for low vitamin D and low albumin circulating levels as strong predictors of worse outcome in COVID-19 patients

**DOI:** 10.1007/s11845-022-02952-9

**Published:** 2022-02-19

**Authors:** Gianfranco Sanson, Amedeo De Nicolò, Verena Zerbato, Ludovica Segat, Raffaella Koncan, Stefano Di Bella, Jessica Cusato, Alessandra di Masi, Andrea Palermo, Pietro Caironi, Pierlanfranco D’Agaro, Roberto Luzzati, Antonio D’Avolio

**Affiliations:** 1 Clinical Department of Medical, Surgical and Health Sciences, University of Trieste, Trieste, Italy; 2 Department of Medical Sciences, Laboratory of Clinical Pharmacology and Pharmacogenetics, University of Turin, Amedeo Di Savoia Hospital, Turin, Italy; 3 Infectious Diseases Unit, Trieste University Hospital (ASUGI), Trieste, Italy; 4 Department of Hygiene and Public Health Unit, Trieste University Hospital (ASUGI), Trieste, Italy; 5 Department of Sciences, Section Biomedical Sciences, and Technology, University Roma Tre, Rome, Italy; 6 Unit of Endocrinology and Diabetes, University Campus Bio-Medico, Rome, Italy; 7 Department of Anesthesia and Critical Care, AOU S. Luigi Gonzaga, University of Turin, Turin, Italy; 8 Infectious Diseases Unit, Clinical Department of Medical, Surgical and Health Sciences, Trieste University, 34123 Trieste, Italy

**Keywords:** COVID-19, Human serum albumin, SARS-CoV-2, Vitamin D

## Abstract

**Purpose:**

We aimed to assess the combined role of vitamin D and albumin serum levels as predictors of COVID-19 disease progression.

**Methods:**

We conducted a prospective observational study on adult patients hospitalized for SARS-CoV-2 pneumonia (March–September 2020). Vitamin D and albumin serum levels were measured on admission. These variables were categorized in albumin < 3.5 or ≥ 3.5 g/dL and vitamin D < 30 ng/mL or ≥ 30 ng/mL. We excluded patients with known bone diseases, renal failure, hypercalcemia and/or treated with antiepileptic drugs and steroids, and patients who received previous vitamin D supplementation. A composite outcome including any ventilatory support, PaO_2_/FiO_2_ ratio, and 60-day mortality was defined.

**Results:**

Sixty-nine patients were enrolled, of whom 50% received non-invasive (NIV) or invasive mechanical ventilation (IMV), 10% died, whereas 89% and 66% presented low albumin and low vitamin D serum levels, respectively. No correlation between vitamin D and albumin levels was found. In multivariable logistic regression analyses adjusted for sex and age-corrected comorbidities, patients having albumin < 3.5 g/dL and vitamin D < 30 ng/mL showed a significant increased risk for all study outcomes, namely NIV/IMV (*OR* 3.815; 95% *CI* 1.122–12.966; *p* = 0.032), NIV/IMV or death (*OR* 3.173; 95% *CI* 1.002–10.043; *p* = 0.049) and PaO_2_/FIO_2_ ≤ 100 (*OR* 3.410; 95% *CI* 1.138–10.219; *p* = 0.029).

**Conclusion:**

The measurement of both vitamin D and serum albumin levels on COVID-19 patients’ admission, and their combined evaluation, provides a simple prognostic tool that could be employed to guide prompt clinical decisions.

## Introduction

Since the beginning of the COVID-19 pandemic, scientists’ efforts have been focused on identifying reliable risk and prognostic factors, aimed at recognizing the most severe form of the disease. Among these, the most represented included older age, pre-existing morbidities (e.g., chronic heart, kidney and lung diseases, diabetes, hypertension, obesity), lymphocytopenia, and elevated C-reactive protein (CRP) and D-dimer, likely highlighting both the inflammatory and coagulator alterations of the disease [1]. Furthermore, both vitamin D and serum albumin deficiency were found as relevant risk factors.

Vitamin D, among its functions, deploys anti-inflammatory effects and a well-established role in innate immunity as well as in boosting adaptive immunity [2–4]. In addition, there are also evidence on a potential role of vitamin D in protecting against acute lung injury or acute respiratory distress syndrome in COVID-19 by targeting the renin-angiotensin system [5, 6]. Vitamin D deficiency has already been associated with worse clinical outcomes in critically ill patients [7, 8]. More recently, low vitamin D levels were found to be associated with increased risk of SARS-CoV-2 infection [9, 10] and worst prognosis, including in-hospital mortality and need for invasive mechanical ventilation [11–13]. For all these reasons, vitamin D replacement has been proposed among different therapeutic options for COVID-19 patients, although its efficacy remains uncertain [14]. Moreover, no consensus exists regarding the definition of vitamin D deficiency and the suggested doses for dietary supplementation. Indeed, whereas several guidelines suggest 12 ng/mL concentration for severe and 20 ng/mL for mild deficiency, more stringent guidelines suggest optimal concentrations over 30 ng/mL [15–18].

The vitamin D concentration is usually determined by measuring the total concentrations of vitamin D metabolites (i.e., 1,25(OH)_2_D and 25(OH)D), assuming that these values indicate the biological available concentration. Only a little portion of vitamin D metabolites (approximately 0.4% of total 1,25(OH)_2_D and 0.03% of total 25(OH)D) are free in the serum from normal individuals [19]. Nearly 85% of vitamin D-circulating metabolites are bound to the vitamin D-binding protein (DBP) and the remaining 15% is bound to albumin.

In critical illnesses such as sepsis, low serum albumin concentrations have been associated with poor outcomes [20]. The association between serum albumin concentrations and disease severity and mortality has been investigated also in COVID-19 patients. The recent literature confirmed the association between hypoalbuminemia, COVID-19 severity [21], and thromboembolic complications [22, 23]. A retrospective study conducted by Kheir et al. found that higher albumin levels on admission were associated with significantly fewer adverse outcomes in COVID-19 patients, including a reduction in venous thromboembolism events, acute respiratory distress syndrome (ARDS) development, ICU admissions, and readmissions within 90 days [24]. Huang et al., for the first time, found out that hypoalbuminemia was an independent predictive factor for mortality in COVID-19 hospitalized patients [25].

A prompt recognition of predictive factors for relevant outcomes is pivotal to mark high-risk populations to provide tailored care. Given the strong prognostic value of low serum vitamin D and low serum albumin on COVID-19 outcomes, we aimed to investigate the potential of combining serum vitamin D and albumin levels to increase the predictive power of the single tests in adults affected by COVID-19 pneumonia.

## Materials and methods

### Study design and population

This is an observational, prospective, longitudinal study enrolling adults (aged > 18 years) with SARS-CoV-2 pneumonia which has been admitted to the Infectious Diseases Unit of Trieste University Hospital, Italy, from March to September 2020.

Patients with known bone diseases (osteoporosis, Paget’s disease, osteomalacia), renal failure with creatinine clearance below 60 mL/min, hypercalcemia (albumin adjusted serum calcium > 10.5 mg/dL), patients treated with antiepileptic drugs and steroids, and patients who received previous vitamin D_3_ supplementation in the previous 3 months were excluded.

The study was conducted in full compliance with the Declaration of Helsinki and the International Conference on Harmonization Principles of Good Clinical Practice. The research protocol was approved by the Ethic Committee (CEUR 2020-OS-072). During the hospital admission, patients signed an informed consent authorizing the use of their anonymized clinical data for research purposes.

### Study outcomes

Both the need for any ventilatory support—either non-invasive (NIV) or invasive (IMV) mechanical ventilation—and 60-day mortality was considered as primary outcomes. Due to the low rate of 60-day mortality, a composite endpoint was thus built, putting together patients who underwent any ventilatory support and/or who died within 60 days from hospital admission.

As a general policy, in the study, hospital patients underwent NIV/IMV in the presence of signs of severe respiratory distress associated to deteriorated gas exchange (according to PaO_2_/FiO_2_ ratio) notwithstanding the maximal inspired oxygen fraction administered (via facial mask or high-flow nasal cannula [HFNC]). However, for each patient the decision of the attending physician to adopt a more-or-less intensive respiratory support strategy was not strictly defined a priori but was established taking into account his/her general clinical conditions and the limitation of the available resources (i.e., ICU beds and mechanical ventilators), also considering prognostic criteria. Consequently, it is possible that patients who could have benefited from NIV/IMV support have been treated in a less aggressive way for the above reasons, with possible impact on mortality. Therefore, we decided to evaluate, as secondary outcome, a marker of poor respiratory exchange, defined as the lower PaO_2_/FiO_2_ value documented during hospital stay. According to Berlin criteria for ARDS definition [26], a PaO_2_/FIO_2_ of ≤ 100 was established to identify a condition of severe hypoxemia.

### Data collection

At ward admission, data on patient’s demographics, past medical history, medications, and further clinical data were collected. To elucidate the impact of co-morbidities, the Charlson comorbidity index was calculated. The age-adjusted Charlson comorbidity index was computed and the burden of comorbidity was considered high in the presence of a threshold of ≥ 5 [27]. Fasting blood samples were obtained in the morning to evaluate complete white blood cell count, platelet count, CRP, albumin, and D-dimer levels. Blood samples were analyzed by standard laboratory techniques, except for CRP that was measured by ELISA. Arterial blood gas analysis (ABG) was performed within 24 h from admission and further ABGs were obtained at different times during hospital stay, depending on the evolution of individual patient’s condition.

Serum vitamin D concentrations were determined at patients’ arrival at the study ward. The analysis was performed through an ultra-high performance liquid chromatography coupled with tandem mass spectrometry detection (UHPLC-MS/MS) method, capable of quantifying in an accurate and specific manner 25(OH)-VD_3_ (mean bias 3.2%, coefficient of variation 4.6%), with a lower limit of quantification of 4.4 ng/mL (range 4.4–150 ng/mL). The same method was capable of discriminating and quantifying 25(OH)-VD_2_, although such a form resulted undetectable in all the tested samples. Calibration standards and stable-isotope-linked internal standards were purchased from Perkin Elmer (Milan, Italy).

A threshold of < 30 ng/mL was chosen as a putative threshold for vitamin D insufficiency in the context of COVID-19, according to previous reports [16, 28, 29]. Findings from a preliminary pilot study performed before starting the present investigation (unpublished data) showed that an isolated vitamin D deficiency could have a greater impact on the study outcomes than isolated albumin deficit. Based on these premises, four albumin-vitamin D categories (AlViD score) were created according to the following categories, taking into account the respective combined levels: AlViD-0 (normal condition), albumin ≥ 3.5 g/dL and vitamin-D ≥ 30 ng/mL; AlViD-1 (isolated albumin deficit), albumin < 3.5 g/dL and vitamin-D ≥ 30 ng/mL; AlViD-2 (isolated vitamin-D deficit), albumin ≥ 3.5 g/dL and vitamin-D < 30 ng/mL; AlViD-3 (associated albumin and vitamin-D deficit), albumin < 3.5 g/dL and vitamin-D < 30 ng/mL.

Patients were followed up until 60 days of hospital discharge, whichever came first.

### Statistical analysis

A minimum required sample size of 37 to 58 patients was calculated a priori to enable a type-I probability error of 5% and a desired statistical power of 90%, based on previous studies adopting a 30 ng/mL vitamin D threshold to document the differences in mortality rate and need for any ventilatory support in COVID-19 patients, respectively [11, 30]. Expecting the risk of lost at follow-up of 5% of the patients to be excluded due to relevant missing data, it was decided to prospectively recruit at least 64 patients.

Data distribution was evaluated using the Kolmogorov–Smirnov test. Continuous variables were reported as medians and interquartile ranges (IQR), while categorical variables as numbers and percentages. Unadjusted comparisons between groups were analyzed via an *χ* test, Fisher test, or nonparametric Mann–Whitney’s *U*-test for independent samples, as appropriate. The bivariate association between continuous variables was investigated using Pearson’s correlation coefficient (*r*). Nonparametric Kendall’s tau-c (*τ*_*c*_) correlation coefficient was performed to assess the strength and the type (positive or negative) of dependence between the AlViD score and the explored outcomes. The strength of the association was interpreted according to the following criteria: < 0.10: very weak; 0.10 to 0.19: weak; 0.20 to 0.29: moderate; ≥ 0.30 strong [31].

The independent association between AlViD-3 score and study outcomes was tested through multiple logistic regression models. Based on a simple rules-of-thumb, the number of covariates to insert in the multivariate analyses was limited to three. Therefore, the logistic models were adjusted for patients’ sex and age-corrected Charlson index high risk category, based on the well-represented association of age, sex, and comorbidities with increased risk of poor outcome in COVID-19. The coefficient of the determination of statistical models was calculated based on the Nagelkerke *R*^2^. The performance of the logistic models in predicting the study outcomes was measured through the *c*-statistic (equivalent to the area under the ROC curve).

Statistical analyses were performed using the software IBM SPSS Statistics, version 24.0 (Armonk, NY, US: IBM Corp.). For all tests, an alpha level of *p* < 0.05 was set for statistical significance.

## Results

### Study population

During the study period, 154 patients were admitted to the study ward and 136 were diagnosed with COVID-19 pneumonia. Sixty-seven patients were excluded according to the above-defined criteria. Accordingly, 69 patients were included in the study.

The main characteristics of the enrolled patients are described in Table 1. The majority of the enrolled population had a low comorbidity index. Only four subjects (5.9%) presented a moderate to severe obesity. The majority of patients had serum vitamin D or albumin levels lower than the normal thresholds (87.0% and 66.2%, respectively). No correlation between vitamin D and albumin levels was found (*r* = 0.161; *p* = 0.191).

**Table 1:** Main characteristics of the study population (*n* = 69)

Variable	Data
Age (years)^§^	72.0; 62.0–79.0
Sex (male)^¥^	46 (66.7%)
Body mass index^§^	25.5; 22.9–28.4
Age adjusted CCI^§^	3.0; 2.0–3.5
Age adjusted CCI ≥ 5^¥^	10 (14.5%)
Hypertension^¥^	42 (60.9%)
Diabetes^¥^	18 (26.1%)
Heart disease^¥^	28 (40.6%)
Active smoker^¥^	5 (7.2%)
White blood cells (cells × 10^3^/mL)^§^	6.1; 4.3–8.6
Eosinophiles (cells × 10^3^/mL)^§^	0.0; 0.0–0.0
Monocytes (cells × 10^3^/mL)^§^	0.4; 0.2–0.6
Lymphocytes (cells × 10^3^/mL)^§^	0.8; 0.5–1.3
Neutrophiles (cells × 10^3^/mL)^§^	4.3; 3.1–7.1
Neutrophil-to-lymphocyte ratio^§^	5.4; 2.8–11.4
Platelets (cells × 10^3^/mL)^§^	211.0; 154.0–291.5
C-reactive protein (mg/L)^§^	88.0; 30.0–131.0
D-dimer (mg/L)^§^	0.7; 0.5–1.8
Albumin (g/dL)^§^	3.3; 2.8–3.6
Albumin < 3.5 g/dL^¥^	45 (66.2%)
Vitamin D (ng/mL)^§^	14.8; 7.4–21.5
Vitamin D < 30 ng/mL^¥^	60 (87.0%)
AlViD^¥^	
Score 0	3 (4.4%)
Score 1	5 (7.4%)
Score 2	20 (29.4%)
Score 3	40 (58.8%)
Higher breathing support^¥^	
Oxygen/HFNC	33 (47.8%)
NIV	21 (30.4%)
IMV	15 (21.7%)
Lower PaO_2_/FiO_2_ ratio^¥^	
> 300	10 (14.5%)
201–300	8 (11.6%)
101–200	16 (23.2%)
≤ 100	35 (50.7%)
60-day mortality	7 (10.1%)

^§^: median; inter-quartile range. ¥: number (percentage). *CCI*, Charlson comorbidity index; *HFNC*, high flow nasal cannula; *NIV*, non-invasive mechanical ventilation; *IMV*; invasive mechanical ventilation

Following hospital admission, all patients required oxygen therapy. Almost half of the enrolled patients presented during their hospital stay a PaO_2_/FIO_2_ of ≤ 100. Overall, 36 patients (52.2%) underwent NIV or IMV, while for the others, the higher respiratory support was oxygen administered via mask or HFNC. The median hospital length of stay was 25 days (*IQR* 15–39). At 60-days follow-up, seven patients (10.1%) were deceased.

### Study outcomes

In the bivariate analysis, patients with a PaO_2_/FIO_2_ of ≤ 100 presented higher serum levels of monocytes (> 100: median 505, *IQR* 330–620; ≤ 100: median 280, *IQR* 180–560; *p* = 0.045), CRP (> 100: median 44, *IQR* 15–97; ≤ 100: median 116, *IQR* 54–171; *p* < 0.001), and D-dimer (> 100: median 0.6, *IQR* 0.4–1.0; ≤ 100: median 1.0, *IQR* 0.5–2.4; *p* = 0.042), a higher neutrophil-to-lymphocyte ratio (> 100: median 3.7; *IQR* 2.5–6.1; ≤ 100: median 8.1, *IQR* 4.6–14.7; *p* = 0.001), and a lower lymphocyte count (> 100: median 1.060, *IQR* 760–1.540; ≤ 100: median 535, *IQR* 400–950; *p* < 0.001).

Patients who underwent NIV/IMV showed a higher BMI (oxygen/HFNC: median 24.1, *IQR* 21.5–27.9; NIV/IMV: median 26.0, *IQR* 23.7–30.9; *p* = 0.023) and serum D-dimer levels (oxygen/HFNC: median 0.6, *IQR* 0.4–1.0; NIV/IMV: median 1.0, *IQR* 0.5–2.8; *p* = 0.025) than patients who just needed oxygen or HFNC. Patients who died within 60 days were older (survived: median 70.5, *IQR* 59.0–78.0; deceased: median 83.0, *IQR* 73.0 94.0 years; *p* = 0.008) and had a higher age-adjusted Charlson comorbidity index (survived: 3.5; *IQR* 2.0–6.0; deceased: 8.0, *IQR* 7.0–9.0; *p* = 0.012) than those surviving.

**Fig. 1:**
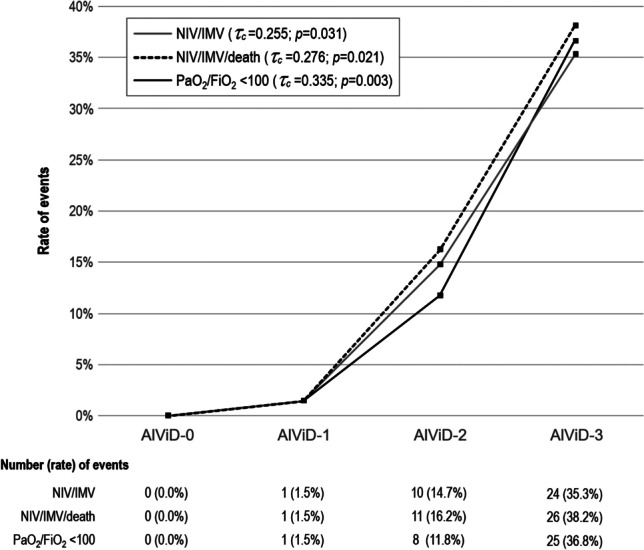
Correlation between the AlViD score and the study outcomes. NIV, non-invasive ventilation; IMV, invasive mechanical ventilation

Vitamin D < 30 ng/mL was significantly associated only with the composite endpoint NIV/IMV/60-day death (normal level: *n* = 2, 22.2%; low level: *n* = 37, 61.7%; *p* = 0.031), while no statistically significant association was found with the other study outcomes (i.e., NIV/IMV and PaO_2_/FIO_2_ ≤ 100). No statistically significant association was documented between low levels of albumin and any of the study outcomes. However, when the respective levels of vitamin D and albumin were combined according to the AlViD score, a statistically significant positive association was shown between a progressive increase in AlViD score and the risk of incurring the adverse events studied. The correlation was moderate for NIV/IMV/60-day death (*τ*_*c*_ = 0.276; *p* = 0.021) and strong for PaO_2_/FIO_2_ ≤ 100 (*τ*_*c*_ = 0.335; *p* = 0.003) outcome. A clear curve inflection point was documented starting from AlViD-2 (Fig. 1).

In multivariable logistic regression analyses adjusted for sex and age-corrected comorbidity burden (Table 2), patients scored as AlViD-3 showed a statistically significant increased risk for all study outcomes, namely NIV/IMV (adjusted *OR* 3.815; 95% *CI* 1.122–12.966; *p* = 0.032), NIV/IMV or death (adjusted *OR* 3.173; 95% *CI* 1.002–10.043; *p* = 0.049) and PaO_2_/FIO_2_ ≤ 100 (adjusted *OR* 3.410; 95% *CI* 1.138–10.219; *p* = 0.029).

## Discussion

Our study showed that, in our population of patients affected by COVID-19 pneumonia, the predictive power of vitamin D and albumin levels on the need for any form of respiratory support and/or 60-day mortality improved when the two markers were considered jointly rather than independently. According to the proposed AlViD score, our data showed that a vitamin D deficiency alone (AlViD-2) identified patients at risk much more clearly than an albumin deficit alone (AlViD-1), while an AlViD-3 condition (i.e., patients presenting both vitamin D and albumin deficiency) strongly marked subjects at higher risk, independently from age, sex and comorbidities.

After more than one year of COVID-19 pandemic, the identification of optimal markers for the early prediction of the severity of the disease, as well as the study of the physiopathological bases explaining the inter-patient variability in the natural history of SARS-CoV-2 infection, is still deserving great interest. In particular, many studies evidenced associations between vitamin D concentrations and SARS-CoV-2 infection, as well as with severity and mortality [32], and among them, some studies even suggest a cause-effect relationship for this association [32, 33]. Currently, randomized clinical trials are ongoing in order to test if vitamin D supplementation could play a role in improving patients’ clinical conditions and reducing mortality from COVID-19; however, results seem not to encourage its use [34, 35]. A link between vitamin D status and COVID-19 incidence and severity could also contribute to explain the seasonal trend of this pandemic (more cases during cold months in most countries), particularly in the northern countries, and the worst prognosis in elderly people, which are known to have significantly lower vitamin D concentrations [36, 37].

On the other hand, serum albumin retains a well-known antioxidant role, other than exerting a significant anticoagulant action: in detail, two recent studies evidenced that hypoalbuminemia is significantly associated with worst COVID-19 prognosis and that albumin supplementation, in preliminary studies, could even play a role in reducing the incidence of thrombotic complications and then, the overall mortality [22, 24, 38].

The complex metabolic interactions between vitamin D and albumin deserve a brief analysis. The fraction of bioavailable vitamin D metabolites is defined as the fraction of free and albumin-bound metabolites. Although the free hormone hypothesis postulates that only the free fraction of hormones is able to enter cells [39], this is not completely true for vitamin D metabolites [19]. Indeed, some organs, such as the lungs and the kidneys, express the megalin/cubilin receptor complex that mediate uptake of DBP-bound vitamin D metabolites [40, 41]. Remarkably, the megalin/cubulin complex is also involved in the endocytotic uptake of albumin in human renal proximal tubule cells [42] and in human epithelial lung cells [43]. It should be also noted that binding of vitamin D metabolites to serum proteins fits a 2-binding-site model: VDBP binds vitamin D with high affinity and relatively low capacity, while albumin has a nearly 1000-fold lower affinity but higher capacity, since it has a 100-fold higher concentration in plasma [19, 44–46]. In normal individuals, the fraction of bioavailable vitamin D metabolites is approximately the 15% of the total circulating vitamin D and comprises the fraction of the free vitamin D and the fraction bound to albumin. As the complexes formed by vitamin D bound to albumin dissociate rapidly, this fraction may be more bioavailable in a dynamically perfused tissue. Therefore, it is likely that the reduction in albumin plasma concentration affects not only the bioavailability of vitamin D metabolites but also their plasma concentration. Notably, in liver disease and cirrhotic patients, the total concentrations and free fractions of vitamin D metabolites correlate to the DBP and albumin concentrations compared to controls [19, 44, 47].

In the current study, we observed that a hybrid categorization of patients based on the combined data about vitamin D and albumin plasma concentrations shows a strongly significant predictive power for all the clinical markers of disease severity, including impaired PaO_2_/FiO_2_, the need for invasive or non-invasive mechanical ventilation, and even mortality. These results could be particularly useful at patients’ admission at the infectious disease wards, in order to identify the patients who have higher risk of experiencing an exacerbation of their clinical condition and, therefore, who could benefit from a more focused and second-line treatment, both against viral replication and inflammation/coagulation, or a more focused monitoring, in order to anticipate any possible detection of further impairment, leading eventually also to admission to intensive care units.

Along this line of reasoning, our study also suggests that a great change in the paradigm about vitamin D “insufficiency” is needed in this context, since the most generally acknowledged definition (vitamin D concentrations < 20 ng/mL for mild and < 12 ng/mL for severe deficiency) [48] is derived from studies concerning calcium/phosphorus homeostasis and bone mineralization. In recent years, the role of vitamin D in the modulation of immune system, with a protective role both against autoimmune and infectious disease, has been evidenced with higher threshold concentration values: therefore, patients with vitamin D concentrations which could be considered normal and sufficient for the prevention of bone diseases could not be enough to protect against infections and inflammatory diseases, such as COVID-19. In detail, in this study, we confirmed that a previously proposed threshold of 30 ng/mL can represent an optimal level in the context of COVID-19, over which a more benign disease evolution is expected, particularly in presence of normal albumins concentration, for patients younger than 70 years old and in absence of serious comorbidities.

Our study has several limitations. First, the sample size is limited; therefore, the number of potential covariates in the multivariate analysis was limited to three, potentially excluding possible other significant independent predictors of disease severity. Second, the timing of vitamin D assessment has been variable among patients, possibly increasing inter-patient variability in vitamin D concentrations. Third, the known capability of systemic inflammation to reduce vitamin D concentrations, due to variations in the concentrations of vitamin D-binding protein and/or modulation of vitamin D metabolism [49–51], prevents us from confirming a univocal cause/consequence relationship between vitamin D concentrations and COVID-19 severity.

In conclusion, the measurement of both vitamin D and serum albumin on COVID-19 patients’ admission, and their combined evaluation, could provide a relatively simple prognostic evaluation that clinicians could take into account (alongside with other multiparametric prognostic scores) both for taking pragmatic decisions and to think over the underlying complex pathophysiological mechanisms that relate vitamin D and albumin depletion with a worse outcome.

## Data Availability

On request.
